# Experiences of central child health services teams regarding a special governmental investment in child health services

**DOI:** 10.1186/s12913-024-11492-0

**Published:** 2024-09-10

**Authors:** Sergio Flores, Anna Sarkadi

**Affiliations:** Department of Public Health and Caring Sciences/CHAP, Uppsala University, Uppsala, Sweden

**Keywords:** Healthcare Policy implementation, Child Health Services, Extended Home Visiting Program, Socioeconomic disparities, Sweden, Agreements

## Abstract

**Background:**

Historically marked by a high infant mortality rate, Sweden’s healthcare reforms have successively led to a robust, decentralized universal child health system covering over 97% of the population 0–5 years. However, inequities in health have become an increasing problem and the public health law explicitly states that health inequities should be reduced, resulting in various government initiatives. This study examines the experiences of Central Child Health Services (CCHS) teams during the implementation of the Child Health Services Accessibility Agreement between the State and the regions starting in 2017. The agreement aimed to enhance child health service accessibility, especially in socio-economically disadvantaged areas, but broadly stated guidelines and the short-term nature of funding have raised questions about its effectiveness. The aim of this study was to understand the experiences of CCHC teams in implementing the Child Health Services Accessibility Agreement, focusing on investment decisions, implementation efforts, as well as facilitators and barriers to using the funds effectively.

**Methods:**

CCHC teams were purposefully sampled and invited via email for interviews, with follow-ups for non-respondents. Conducted from January to October 2023, the interviews were held digitally and recorded with individuals familiar with the agreement’s implementation within these teams. Both authors analyzed the transcripts thematically, applying Braun and Clarke’s framework. Participants represented a cross-section of Sweden’s varied healthcare regions.

**Results:**

Three main themes emerged from the thematic analysis: “Easy come, easy go,” highlighting funding uncertainties; “What are we supposed to do?” expressing dilemmas over project prioritization and partner collaboration; and “Building castles on sand,” focusing on the challenges of staff retention and foundational program stability. Respective subthemes addressed issues like fund allocation timing, strategic decision-making, and the practical difficulties of implementing extended home visiting programs, particularly in collaboration with social services.

**Conclusions:**

This study uncovered the challenges faced in implementing the Child Health Services Accessibility Agreement across different regions in Sweden. These obstacles underline the need for precise guidelines regarding the use of funds, stable financing for long-term project sustainability, and strong foundational support to ensure effective interprofessional collaboration and infrastructure development for equitable service delivery in child health services.

## Background

Sweden’s child healthcare is repeatedly reported as very good in international standings. United Nations International Children’s Emergency Fund (UNICEF) and the Organisation for Economic Co-operation and Development (OECD) score Sweden highly on several indicators, such as immunization, child mortality, and infectious diseases [1, 2]. The Swedish Child Health Care Register reports that in 2021, child healthcare reaches over 97% of all children in Sweden. Although voluntary, virtually all children under six have had contact with a child healthcare center [3].

Sweden’s relatively high child healthcare coverage and quality standards culminate decades of progressive policy reforms and increased democratic participation in healthcare decision-making. Over time, increased public and civil involvement in policymaking heightened concern for vulnerable groups, including children, and expanded individual rights, emphasizing the right to health for adults and children [4]. While child healthcare in Sweden during the 1960s focused on monitoring and tracking disabilities, by the 1970s, it shifted towards fostering holistic child development [4]. In 1980, the Swedish Parliament mandated parental education in maternal and child healthcare to improve family conditions, enhance parental knowledge, and influence societal conditions for children [5].

This democratic process created a complex healthcare policy landscape with multiple key players. The Ministry of Health and Social Affairs oversees national healthcare policies, aided by about fifteen agencies, including the National Board of Health and Welfare and the National Public Health Agency. Regionally, 21 councils and bodies fund and provide healthcare, while the 290 municipalities serve the elderly and disabled. The Health and Medical Services Act of 1982 designates county councils/regions and municipalities responsible for ensuring access to quality healthcare for all residents in Sweden [8]. While regions manage and finance healthcare locally, the government mandates national consistency and equal access to care for all citizens, regardless of identity or location, through overarching health policies, regulatory frameworks, and national health guidelines.

Additionally, the state can stimulate certain areas or goals of healthcare through dedicated funds, providing financial incentives for regions to prioritize specific health initiatives. The regions and municipalities are represented by the Swedish Association of Local Authorities and Regions (SALAR), which negotiates and facilitates agreements with the government regarding these special governmental funding packages. This ensures that the allocation and use of funds are aligned with national health priorities while addressing local needs [7].

### Agreements within Swedish governance

In Sweden’s governance structure, agreements between the government and the Swedish Association of Local Authorities and Regions (SALAR) exemplify the interplay of various governance theories. These voluntary agreements, central since 1985 especially in healthcare, are characterized by performance-based funding for regional and municipal projects. Despite their voluntary nature, they exert substantial normative and financial pressure on local politicians, as emphasized by the Swedish National Audit Office in 2014 and 2017, challenging the notion of non-participation and complicating governance roles [6, 7]. Marcusson views SALAR’s involvement as enhancing municipal autonomy. However, it also creates a complex dynamic with the National Board of Health and Welfare, leading to potential conflicts and challenges in coordinating healthcare policies and implementation [8].

These agreements epitomize ‘soft governance’, contrasting with the ‘hard governance’ of legal regulations. Sweden prefers negotiations and consensus, which has a long tradition in Sweden and also aligns well with the EU’s Open Coordination Method [9]. Multilevel governance theory describes a decentralized, networked structure focused on non-hierarchical, negotiated interactions [10–12]. Polycentric governance complements this, advocating for multiple overlapping, autonomous decision-making centers, reflecting Sweden’s decentralized yet interconnected governance [13, 14]. This dual approach, balancing autonomy with cooperative negotiation, demonstrates Sweden’s ability to use various governance tools to maintain a dynamic, yet cohesive system.

### Agreements in the context of good care close to the patient

After an Effective Care report in 2016, Sweden initiated the “Good and Close Care” policy transition in 2017 [15]. This shift aimed to enhance healthcare resource efficiency by expanding primary care and reducing reliance on hospital-based care, in line with current and future sociodemographic needs. A commission was established in 2017 to explore and improve healthcare efficiency and resource allocation. This commission’s ongoing work and the general momentum around the need for enhanced collaboration, continuity, and person-centeredness in healthcare led to the State and SALAR entering the Child Health Services Accessibility Agreement for 2018–2020. 120 million SEK in state grants were allocated to strengthen child healthcare, with plans for annual agreements over three years. In 2020, the commission’s final report (SOU 2020:19) further emphasized the importance of these collaborative structures and the need for sustained efforts in this direction [16, 17]. Due to government formation delays after the September 2018 elections, the 2019 agreement was finalized in May 2019, and state subsidies followed. Although the agreement covered three years, funds had to be formally allocated each year after a budget decision in parliament. Hence, the regions were not automatically guaranteed or received funds for three years or yearly on a predetermined date, causing uncertainty.

The Child Health Services Accessibility Agreement stated the following: *“The primary objective is to enhance the accessibility of child healthcare for groups with suboptimal health*,* dental health*,* and lower vaccination rates by bolstering home visits or other outreach activities*,* especially in socio-economically disadvantaged areas with potential low adherence to care programs.”* [17]. However, there was no clear definition as to what exactly was meant by “accessible services” and consequently no set goals to achieve. In a subsequent report detailing the agreement’s implementation, the Swedish National Board of Health and Welfare defined it *as " targeted interventions are developed and to a greater extent provided according to the need and adapted to the varying conditions on children and parents”* [17]. Investing in services in socioeconomically vulnerable areas was not specifically mandated, leaving room for interpretation of both the goals, the target groups, and the means of governmental investment, as pointed out below.

### Home visits

Home visits were emphasized in the agreement to support socioeconomically vulnerable children, a tradition dating back to the early 20th century. The National Board of Health and Welfare’s 1991 guidelines recommended offering new parents a home visit within five days of leaving maternity hospitals [18], and current guidelines from the National Handbook of Child Healthcare stipulate a minimum of two visits: one within the next first month, and the second at eight months [19]. The agreement referenced the promising extended home visiting program in Rinkeby from 2013 to 2015, which provided first-time parents with four additional visits (for a total of six) by a child health nurse and a social services parent advisor for children aged 0–15 months. It is noteworthy that no randomized controlled trial of the Rinkeby home visiting program had been conducted at the time: the initial positive results were based on qualitative and quasi-experimental studies in one region [20–23]. Although promising, neither of these studies qualified for evidence-based decision-making.

### The regional child health services as receivers of special government funds

The regional councils are elected bodies responsible for various public services, including healthcare, education, and public transport [24]. A total of 21 autonomous regions are organized into six larger healthcare regions for certain aspects of specialized care and care guidelines. Child health services are provided by child health nurses at primary healthcare centers in each region, supported by a family physician locally and a regional Central Child Health Service (CCHS) team. The latter comprises pediatricians, nurses, psychologists, and other experts to oversee the contents of child health services and children’s health at the regional level. CCHS teams provide consultations, training, and implementation support and ensure adherence to national guidelines, serving as reference points for regional and national child health directives. They collaborate with various local, regional and national stakeholders [19].

The agreement between SALAR and the government encouraged regions to use government funds to enhance their child health service development. A 2020 national survey by The National Board of Health and Welfare revealed disparities in the pace and scope of activities implemented across regions responding to the special government funds [25]. Some regions learned of the first agreement in December 2017 through SALAR, while teams learned of the agreements later in 2018, and even those informed earlier encountered delays in grant utilization decisions, pushing planning to late 2018 [25]. The National Board of Health and Welfare’s report also highlighted differences in regional strategies, with some regions quickly utilizing grants to enhance existing initiatives. In contrast, others faced challenges due to the absence of prior special initiatives [26].

The National Board of Health and Welfare’s mapping also found variations in regional planning and management resources for the agreements, including disparities in CCHC team staffing and timelines for grant utilization. Many regions lacked sufficient CCHC team staffing for prompt grant handling, necessitating resource allocation adjustments. CCHC team sizes ranged from one to almost 17 full-time positions, implying very different capacities to manage new funds or initiatives. Thus, although CCHC teams were not the primary intended recipients of special government funds (the regions were), The National Board of Health and Welfare’s survey showed that CCHC teams had a central role in distributing and managing these funds.

## Objective

To understand the experience of CCHC teams in implementing the Child Health Services Accessibility Agreement between the Swedish state and SALAR regarding investment decisions, implementation efforts, as well as facilitators and barriers to using the funds effectively.

## Methods

### Interviews

A convenience sampling method was used to select participants based on the official contacts listed on each region’s CCHC webpage. Within each CCHC, we aimed to interview key informants knowledgeable about implementing the Child Health Services Accessibility Agreement. This typically included senior child health nurses, project coordinators, and team leaders overseeing child health services in their regions. The participants were recruited by email sent to all CCHC teams in Sweden. Together with the invitation to participate, a summary of the project, the ethical approval, a request to schedule an interview, and a copy of the interview guide (Table 1) were sent in advance to allow time for reflection. If there was no response, another email was sent two weeks later. A PhD student who speaks only basic Swedish conducted individual interviews in English from January 2023 to October 2023. The interviews took place via Zoom or Microsoft Teams, were recorded in digital audio, and lasted an average of 35 min. To ensure the validity and reliability of the data collected, participants were allowed to use Swedish terms where necessary to express specific concepts accurately. Additionally, all transcripts were reviewed by bilingual researchers to verify the accuracy of the translations and interpretations.

**Table 1:** Interview guide

Questions
1. In which region do you work?
2. Which Child Health Centers do you oversee (large regions sometimes have multiple C-CHS sub -teams)?
3. At what level was a decision taken on how to use the state funds in your region?
4. Was a strategic decision made only to involve certain child health centers (e.g., low socioeconomic status), or was the money generally used to strengthen the whole child health service system?
5. Which projects were implemented in your region between 2018–2020 with the Child Health Services Accessibility state funds? (Choices were presented to the respondent)
6. If none of the options in the previous question is a project you recognize as funded by the 2018–2020 state grant, what did you do instead?
7. If your child health services implemented an extended home visiting program, could you please answer the following: a. Did the program have a specific name and/or follow a manual? b. How many visits did your program include to each family? c. When were the first and last visits done? d. How often would these visits occur? f. Which actors were involved in these extended home visiting programs?
8. Would you say the project(s) funded by the 2018–2020 state grants were (was) a new intervention or an extension/expansion of an existing intervention?
9. How were the children, families, or groups that benefited from the project identified? Please describe any criteria used (i.e., socioeconomic indices, geographic location, other sociodemographic parameters, etc.)
10. When did this funded project start to operate (regardless of it being a new project or an extension of an existing one)?
11. Is the project still ongoing?
12. If the previous answer was positive, how is it funded after the state funds stopped? Tell me more about the region’s thinking on this matter (or indicate who might be good to talk to)
13. How much money did your region spend to implement the state-funded project yearly?
14. What percentage of those costs were covered by the state grants?

Twelve CCHC teams agreed to participate in interviews; one declined to participate, two answered our interview guide in written form, and the rest did not reply (Region Kronoberg, Region Kalmar, Region Värmland, Region Gävleborg, Region Blekinge, Region Norrbotten). In total, 21 CCHC teams were contacted. The sample included regions from all six major healthcare regions, encompassing areas of different sizes and profiles regarding urban versus rural and varying percentages of immigrant populations.

### Data analysis

The interviews were recorded, and the interviewer took detailed notes. A research assistant then transcribed the recordings, which one of the authors reviewed.

Both authors coded the interview data. The data were analyzed based on Braun and Clarke’s approach to thematic analysis. The initial step in our process involved identifying broad themes from the material. These themes were then further refined and divided into more specific categories. This approach allowed us to examine the material for overlaps or commonalities within these categories, leading to a more focused and organized analysis. The process involved continuously refining the themes and categories, ensuring each category was well-defined and aligned with the overarching themes. This iterative process continued until we reached a point where no new themes or insights emerged from the data, a stage commonly referred to as data saturation in thematic analysis. Achieving data saturation ensured that our interpretations were comprehensive and accurately reflected the participants’ experiences.

Sergio Flores, a man with a background in public health and experience in interdisciplinary projects and mixed methods studies, conducted interviews, led data analysis, and contributed to the interpretation of findings. Anna Sarkadi, a woman with extensive experience in qualitative research in child health services, designed the research framework, assisted in data coding and analysis, and contributed to the interpretation of findings.

## Results

Three themes emerged during the analysis: *“Easy come*,* easy go”*, *“What are we supposed to do?”* and *“Building castles on sand”*. These were further divided into subthemes (Table 2**).**

**Table 2:** Results from the thematic analysis

Themes	Subthemes	Description
“Easy come, easy go”	*“Timely Arrival of Funds”*	Uncertainty on when funding would start after the signing of the agreement.
	*“Can I plan around these funds?”*	Uncertainty on how far ahead they could count on funding their projects.
“What are we supposed to do now?”	*Project Prioritization Dilemma*	Initial unclarity on which kind of project they should support with additional funds.
	*“Sorry*,* wrong address?”*	Differing criteria on how the project beneficiary population would be selected.
	*“Who should I partner with?”*	Challenges involving other actors necessary to implement extended home visits.
“Building castles on sand”	*Staff quicksand*	Great difficulty in hiring or retaining qualified staff to perform home visiting programs.
	*Weak foundations*	The two home visits that are part of the national program need to be fulfilled before moving to extended home visiting programs. The program also depends on a system of resources to which parents can be referred, such as health care, social services, debt counseling, etc.

Table 3 provides information about the respondents’ general locality and region, categorized by population density, location, and urban-rural distinctions.

**Table 3:** Respondent information

Source	Description
A	High-density urban center (Southern Sweden)
B	Medium-density urban area (Eastern Sweden)
C	Medium-density rural area (Central Sweden)
D	High-density urban center (Western Sweden)
E	Low-density rural area (Island region)
F	Low-density rural area (Northern Sweden)
G	High-density urban center (Southern Sweden)
H	Medium-density rural area (Central Sweden)
I	Medium-density urban area (Eastern Sweden)
J	Low-density rural area (Northern Sweden)
K	Medium-density rural area (Central Sweden)
L	Medium-density rural area (Central Sweden)

### “Easy come, easy go”

#### Can I plan around these funds?

The source of the funding received by the CCHC teams was the Child Health Services Accessibility Agreement between the Swedish state and SALAR and represented a relatively easy acquisition of funds. Still, its temporality made it difficult for regions to plan their investments with a long-time horizon and many interviewees highlighted their reluctance to engage in long-term planning. This reluctance was also sometimes apparent on the regional level due to a lack of sustainability.

*I think in general*,* our lead in the region is in some way afraid of using that kind of money because it’s difficult to continue after the money is stopped*,* yes. So*,* I heard in discussions that they are afraid of funding from SALAR*,* for example*,* because it’s difficult to find that money after. (Low-density rural area)*

Interviewees described the state funding as temporary support and were uncertain about what would occur once the agreement ended. This wielded a pivotal influence on the kind of investments they chose to do. Participants indicated that the uncertainty prevented them from allocating funds to projects demanding substantial investment, organizational dedication, or ongoing expenses, such as extended home visiting programs.

*Uh*,* it’s always a problem with these state funds that come for one year*,* you don’t know what happens next year because… how should you plan? You don’t know; this home visiting program goes for up to two years of the child’s life cycle*,* and you have to hire personnel and you can’t hire like… okay*,* you can work here to this year then we don’t know*,* if you’re lucky*,* you get to work next year. It’s a big problem with these short funds we think. (Low-density rural area)*

This hesitation was noted as a reason why some regions prioritized funds for personnel training or infrastructure enhancement. In certain cases, regions that chose to direct funds toward extended home visiting programs, despite the prevailing uncertainty, did so with modifications.

*[We] targeted money for home visits together with the*,* with the social services (…) but we did not make a full Rinkeby model home visiting program. (Medium-density rural area)*

Some interview participants also provided examples of the lack of sustainability after the initial funding, hence the term “easy-go” in the theme name.

*Right now*,* we don’t have an extended home visiting program (…) [Name of place] started a few years ago a model like the Rinkeby model but not as many home visits as the Rinkeby model. But now the money is ended*,* and they have decided not to continue with that program. (Low-density rural area)*

However, one of the larger regions, with greater organizational and human resource capabilities, managed to strategize around a three-year timeframe. It’s worth mentioning that this region had regionally decided strategic funds prior to the state funds to prepare for the implementation and eventually sustained the extended home visiting program with continued regional funds after the agreement concluded.

#### “Timely arrival of funds”

Some interviewees noted that the initial distribution of funds was inconsistent and challenging to strategize around.

*If we had known that next year (…) you will have money*,* then we could start the project*,* you know*,* without any costs*,* but prepare everything until the day that the money came and then we could use the money immediately for the*,* so that*,* because it was*,* we had them so late. So*,* the first year’s money*,* we couldn’t use them at all because it was so late. (Medium-density rural area)*

This inconsistency made it difficult to prepare the necessary human resources and operational capabilities for projects like the extended home visiting program and even for pilot initiatives.

*Yeah*,* no*,* yes*,* you can say at the end of 2018 we got the money really late*,* you know*,* in the autumn*,* you can say. And so*,* it has*,* takes a little time to start it up. So*,* the nurses started to work in 2019. (Medium-density rural area)*

### “What are we supposed to do now?”

Another area of concern was the unclear guidelines regarding the goal of the funds, the interventions that should be prioritized and which partners to use, reflected by the three subthemes: *Dilemma in project prioritization*, *Sorry*,* wrong address*, and *Who should I partner with?*

#### Dilemma in project prioritization

It was unclear whether or not the decisions regarding the state funds should be made on a regional/political level or by the CCHC teams. Were the decisions to be based on political will or professional opinion?

*Yes. I think when you are targeting in child health care*,* you should have conditions saying that this money*,* should go to those who work with child health care development. This time it didn’t*,* so it just went past us by*,* to other people who were ambitious*,* and they had ideas (…) so I would like to see that. Some conditions for who should take care of this. (High-density urban center)*

Sometimes the decision was a result of a dialogue between these two levels.

*That we could say that we from CCHC teams see that this is something that we need to do to make it better for the children that come visit us and make it better for them in the future and for the health and so forth*,* and that we had that trust from our region that ‘you know what you’re talking about’*,* and you know what the units need*,* and what the children need*,* and what their parents need. (Low-density rural area)*

At other times, the allocation was based on an application procedure.

*We wrote up a proposal for [Name of the region]*,* for the*,* to use the governmental funds. Um*,* and it was so the it was um*,* it was a political decision. To allow us to use the funds within the confines of a project. (Medium-density urban area)*

The participants also described the basis on which to make resource allocation decisions as rather unclear, sometimes resulting in simply distributing funds evenly to child health service units.

*In our region*,* we were given*,* 11 different child health care units were given the money and they were*,* they could do whatever they liked with the money… (Medium-density urban area)*.

Some regions felt that allocating funds to individual child health units was almost unfair, given their lack of competence in project management.

*Then it’s hard to say*,* here you have some money and do something. Because then you don’t have*,* no*,* most*,* a lot of them don’t have like the competence in making an implementation… or making a project plan (…) So*,* I don’t think it was kind of fair. (Medium-density urban area)*

Nevertheless, various projects were launched in several regions, each achieving different progress and outcomes. Some regions started extended home visiting programs, with some closely following or expanding upon the Rinkeby model.

*We knew what we wanted to do and how we could do it since we had Rinkeby as a good example. And we also had the benefit that we had two units that were interested and I had a good staff that made it possible for them to conduct the home visiting. (High-density urban center)*

Others expanded the conventional two-visit program by adding more visits or other staff to the child health nurses. Others ventured into distinct projects like the Very Important Babies model for work with high-risk families experiencing substance use, violence, mental illness, or cognitive disabilities; the Swedish version of the Safe Environment for Every Kid psychosocial risk screening model; the Circle of Security attachment enhancing program; child health teams for collaboration across organizations, and oral health initiatives. Some regions mainly channeled their funds into enhancing health centers, procuring new equipment, or training staff.

#### Sorry, wrong address

The decision regarding the target group of the agreement’s interventions was another recurrent theme in our interviews regarding unclarities related to funding allocation. It did not seem entirely clear to the participants whether the funds would be distributed to all child health services to promote universal interventions or only used for selected areas.

*The only problem was that we didn’t see… The National Board of Health and Welfare*,* they should have said that this is for people that need it the most. Because now it was too free*,* like that’s why this project fit in for everyone. (High-density urban center)*

In striving to achieve the stipulated goals of the agreement to target those who *“have poorer health and dental health and lower vaccination coverage”*, the majority of regions utilized the Care Needs Index, a tool designed to gauge the health risk of a population, and often a key factor in determining healthcare reimbursements [27]. Yet, there wasn’t a uniform methodology across regions. Some employed distinct socioeconomic indices, while others relied on indicators like crime rates and prevalence of maternal smoking and tuberculosis to pinpoint their target groups.

In certain regions, politicians actively participated in deciding which areas to include.

*Politicians were very interested in targeting some places that were high in crime and social problems. (High-density urban center)*

#### Who should I partner with?

A recurring concern among our interviewees was the challenge of implementing the extended home visiting program, which required child health services to partner with social services. The home visits would be conducted in pairs with a nurse and a social worker to achieve its goals effectively. The capability to form and work within such teams appeared to be restricted due to funding and organizational constraints.

*Yeah*,* and how*,* because the problem now is that the municipality is*,* which is not a part of the region*,* doesn’t want to invest in social care. And then you don’t have the money*,* and then you don’t have the persons*,* and then you can’t have a home visiting program with the social care. (Medium-density urban area)*

Several regions conveyed that the allocated funds were earmarked strictly for healthcare expenditure. While these funds facilitated the hiring of nurses, integrating or retaining social workers proved difficult.

*And so*,* the social worker also got a room at the*,* beside the nurses*,* and she could sit there and go out to all the families there. But then*,* because (…) they only provided money for the nurses because this*,* this money was for the health care (…) …. so*,* she couldn’t go with the nurse to the home visiting program and the nurse was very much alone and she was*,* she quit. (Medium-density rural area)*

As evident above, this often also directly impacted the sustainability of the extended home visiting program and participants commented on the need of funds to be allocated to all parties involved in implementing an intervention.

*And also*,* money allocated to all parts of the cooperation*,* not only to*,* of course we will want money for the child health care*,* but also for the partners*,* otherwise it’s a bit tricky. (Medium-density rural area)*

Nevertheless, some of these regions found solutions to use the funding to promote collaboration.

*And so*,* in certain areas they did*,* the nurses brought the social services*,* or the social services joined the nurses at home visits for newborns. That is already in the program*,* basic program*,* but they made it together*,* that first home visit*,* and then we supported that*,* or the region did by 2000 SEK per this kind of home visit*,* that was together with the social services. (Medium-density rural area)*

Collaboration with dental services was also highlighted as a valuable component to provide the “extra dose” of home visiting for families in need.

*And also*,* we said before about the dental services*,* it was also included*,* or catalyzed by this money and at one family centers*,* they do visits together. They have those visits*,* the dental services and the nurses at 10 months. (Medium-density rural area)*

However, it was often mentioned that being under a different administrative unit adds a layer of difficulty to incorporating dental services, despite available funding.

*The collaboration with the dental clinics*,* we have not been able even if they have received money every year from the funds*,* (…) but they have not extended their work. (High-density urban center)*

To improve collaboration, some regions proactively helped staff prepare for the roles together in the new home visiting program.

*Uh*,* we let them*,* the nurses and the social worker*,* go away for a lunch-to-lunch meeting*,* for example*,* to try to find out how to collaborate in a better way*,* and then we could use the money to have someone else to come and support them (High-density urban center)*.

In one successful example, the municipality allocated funds to match the need for social workers collaborating with nurses.

*So*,* in*,* in one municipality that has*,* according to CNI and child poverty*,* very great needs - then the municipality made an investment*,* so they allocated their social worker to the family center*,* and we could initiate a four-occasion extended home visiting program*,* where all families who have their first child or the first child born in that municipality*,* they receive four home visits from the social worker and child health care. (Medium-density rural area)*

### “Building castles on sand”

Regions displayed a wide range of regional planning and resource management capacities and existing child healthcare initiatives well before the agreements were signed. That conditioned if extended home visiting programs could be implemented, either facilitating or hindering it. We grouped the subthemes in two:

#### Staff quicksand

Virtually every region expressed some concern in finding and maintaining personnel resources when implementing extended home visiting programs in a way that reaches the intended effect. A common point was the difficulty regarding getting skilled personnel to apply for jobs in disadvantaged areas and staff turnover due to uncertain employment conditions or heavy workload. This was the case even before implementing an extended version of the “standard” two home visits as laid by the National Child Healthcare Program. Therefore, some regions used the funds to “catch up” with national standards concerning nurse staffing and home visits.

*So*,* you can see that we have*,* we have to start from the basics*,* from the beginning*,* and to get everybody to do their home visiting at*,* before four weeks*,* and then at eight months. (Medium-density urban area)*

Many regions expressed in one way or another that due to the uncertainty in the availability of funds and the difficulties in coordinating with other personnel, the workload was such that staff would quickly take sick leave or quit, jeopardizing any nascent attempts to implement extended home visiting programs.

*It’s a small city*,* so they were one and a half nurse*,* and then that half nurse quit and left the town and the social worker left so*,* the one nurse was all left alone and of course she couldn’t do the work and she was extremely*,* she was*,* what can I say*,* disappointed because they have done such a good work and now there was nothing. So*,* she quit because she couldn’t have the strength to keep up. (Medium-density rural area)*

#### Weak foundations

Many regions thought that conditions in their communities were inadequate even to attempt an extended home visiting program. As mentioned before, some centers believed that they were lagging on the two-home visit standard. Even child health care infrastructure was brought up as not being adequate.

*A subproject with extended home visits according to Rinkeby*,* at a child health unit with high care needs*,* based on CNI (care need index) in collaboration with the social services in S municipality. The health center in question*,* which the unit was part of*,* was closed in the autumn of 2019. The offer to participate in the project was made to a nearby child health unit*,* but they declined as a new nurse had just been recruited. A decision was made to discontinue the project*, as there were no conditions for implementation. **.

**Written response, no interview // (medium-density rural area)

Others pointed out that in the child health centers that were physically co-located with a Family Center, collaboration logistics were most optimal.

*But what happened mostly was that*,* mainly family centers in certain areas where child health nurses and social services already were placed together*,* were working together. It was easier for them to start making home visits together as well. (Medium-density rural area)*

A concern that was brought up across several regions was the lack in technical preparation from administrative and operational teams meant to be involved in extended home visiting programs from the start. Some regions spent time and money training and aligning different personnel before the beginning of the project.

*I think that the money sort of catalyzed this cooperation so it made it easier to focus on that*,* and also to have these workshops and trainings*,* and things together. And we also did some evaluation of this cooperation for this money that we got. Like we evaluated the family centers*,* for instance*,* and also*,* the cooperation with the dental care. So*,* we were eight people*,* you could say*,* who were in charge. (…) There were so many different questions. They didn’t know how to you know*,* like for instance*,* having a system where they could book the [home visits]. So*,* that was one issue. But then they (…) needed to team with each other. So*,* every group of home visiting staff*,* they would have like*,* us*,* we organized a kick-off. (High-density urban center)*

Others felt that the managerial skills necessary to implement projects could not be expected from personnel trained as clinicians, and they did not have access to or did not think of asking for another kind of project assistance.

*Because we’re trained in medicine*,* right? We don’t know how to manage projects. That’s a big*,* big lack for us. (Low-density rural area)*

Other regions pointed out that leadership structures are important in the success or otherwise of the project implementation.

*The leadership is important in implementation*,* like the different bosses. And it’s always difficult when there’s two different bosses involved*,* one for the social worker*,* one for the child health center. But they weren’t involved enough. (High-density urban center)*

Yet, regions that did put implementation support in place wondered about sustainability.

*I mean a lot projects can work well for a while*,* when there’s project funding and people think it’s fun*,* and new*,* but then when you don’t have extra money and it’s not fun and new anymore*,* if you haven’t had the support to make it work (…) then it’s then it’s probably not going to continue working. (High-density urban center)*

## Discussion

This study uncovered the challenges faced in implementing the Child Health Services Accessibility Agreement across different regions in Sweden. The three main themes highlighted funding uncertainties, dilemmas over funding goals and definitions, project prioritization and partner collaboration, and the challenges of staff retention and foundational program stability. Respective subthemes addressed issues like fund allocation timing, strategic decision-making, and the practical difficulties of implementing extended home visiting programs, particularly in collaboration with social services. These issues highlight the complex relationship between financial, organizational, and human resource factors that influence decision-making and the implementation of interventions across Swedish regions.

### Ambitious and welcome, but ill-defined

The agreement between the regions and the central government was ambitious in its intent, but left ample room for interpretation and gave no guidance to regional decision-makers. An ambitious scope also makes execution more complex, as healthcare projects, especially those targeting vulnerable populations, often face challenges at both policy and implementation levels [28–30]. Under these circumstances, clear definitions and guidance are key. Yet, all of the central concepts of the agreement were left undefined: “The primary objective is to enhance the *accessibility* of child healthcare for *groups with suboptimal health*, dental health, and lower vaccination rates by bolstering *home visits or other outreach activities*, *especially in socio-economically disadvantaged areas* with potential low adherence to care programs.” The ambiguity perceived by the recipients of the funds was highlighted in the theme “What are we supposed to do now?”. More clarity in definitions of the main outcome, accessibility, as well as target groups would have led to less frustration and less varied implementations across regions.

This ambiguity echoes recommendations by WHO, who emphasized that while flexibility can foster innovation, too much latitude without guidance in an organization without the skills or resources for implementation can lead to scattered efforts and reduce the impact of an initiative [31]. The varied projects that emerged—ranging from replicating or modifying the Rinkeby extended home visiting model to other standardized models or programs or local initiatives or even just general resource enhancement at times—reflect the regions’ efforts to interpret and act upon the agreement’s broad directives. The inherent ambiguity of the agreement and the subsequent interpretation by The National Board of Health and Welfare led to different targeting approaches. The regions’ choice to target vulnerable populations could have been influenced by defining ‘poorer health outcomes’ and providing a clear rationale why regions should address socioeconomically disadvantaged areas primarily, along with suggestions of how these might be defined. As it turned out, several regions did target socioeconomically vulnerable areas, whereas others spent funds across all child health units and others again avoided or failed to implement projects in difficult areas due to staff shortage, collaboration difficulties or lack of implementation support.

### Funding uncertainties

The “Easy come, easy go” theme reveals critical funding uncertainties. Regions faced the challenge of not knowing when they would receive the funds or how much would be allocated to their projects, and for how long. Such unpredictability in funding has been recognized as a significant barrier to long-term project planning [32]. Without a reliable funding schedule or clarity about fund distribution, regions found it challenging to strategize and allocate resources effectively. These funding challenges parallel the observations made by McDaid and Park, who argued that inconsistencies in financial allocation could lead to project failures or hinder project scalability, and that this is a frequent issue affecting organization’s sustainability when reliant on delegated financing [33].

Criticism has been levelled against the use of state funds to achieve goals within certain specified areas of care previously [34]. Specifically, billions have been spent by the state on trying to affect psychiatric care, with little or no evidence of their efficacy in achieving desired outcomes. Critiques argue that due to the unreliability and short-term nature of these funds they cause more disruption in service provision than they provide possibilities for improvement [34]. Short notice, the mandate to spend money within a restricted funding period and no long-term availability to plan for make these funds inefficient in improving service delivery.

Yet, allocating state funds for specific purposes is a powerful political tool, displaying a willingness to invest money and ability to act in response to raised concerns in healthcare. Thus, chances are state funds will be used in the future as well. Therefore, it is the responsibility of SALAR, the representative agency for municipalities and regions, to secure agreements with sufficient decision support, definitions, targets, funding time frame and payment dates so that regions have a chance to use funds adequately.

### Services are made of people

Our findings also highlighted the importance of inter-professional collaboration in implementing extended home visiting programs, aligning with studies asserting that multidisciplinary teams are essential for holistic healthcare delivery [35, 36]. A paper looking into Home Visiting Programs in Sweden reached a similar conclusion from a middle manager’s point of view [37]. However, structural and organizational constraints appeared to be significant hindrances. The administrative friction between healthcare and social services departments, and even within the healthcare sector itself, revealed compartmentalizing that impede collaborative efforts. The challenge of incorporating social workers into these programs is a testament to these systemic barriers. Yet, it is also worth noting that Family centers made such collaborative efforts much simpler.

The “Building castles on sand” theme underscored foundational challenges. This name aptly reflects the sentiment that extended home visiting programs, as promising as they may be, require a stable foundation to succeed. Factors like staffing shortages, high turnover rates, and inadequate infrastructural readiness have been recurrent themes in healthcare implementation research [38, 39]. Our findings reiterate the importance of ensuring that basic prerequisites, like sufficient staffing and appropriate training, are met before embarking on ambitious projects. The difficulties in maintaining trained staff, highlighted by many regions, echo the global concern of healthcare worker retention [40, 41].

Despite the challenges highlighted in implementing the Child Health Services Accessibility Agreement, some regions had better success in utilizing the funds effectively. Strategic planning and preparedness, such as having prior plans in place, allowed for swift mobilization of resources. Pre-existing and fluid interprofessional collaboration between child health services, social services, and other stakeholders enabled better integration of services as intended by the agreements.

These observations could better guide future funding allocations. Investing in capacity building for project management and leveling the regions’ starting conditions could significantly improve the chances of success. Regarding the funding itself, sustainable models with multi-year commitments and robust monitoring and evaluation frameworks are recommended to ensure long-term planning and continuous improvement.

### Methodological reflections and limitations

Credibility was achieved by using a semi-structured interview format, blending structured questioning with space for spontaneous insights. Participants’ prior access to interview questions likely enriched responses, and a consistent interviewer ensured minimized biases. Data authenticity was further underscored through transcript review. A potential threat to credibility was the fact that the interviews were conducted in English which was not the respondents’ native language. However, allowing participants to mix Swedish in their responses helped them correctly express specific terms. Member checking was not offered in this study, which we acknowledge as a limitation.

The study’s dependability was achieved through careful documentation of the data collection process and the adoption of Braun and Clarke’s thematic analysis. Dual-coding by both authors enhanced the dependability of findings.

To reach confirmability, our methodological transparency, especially in sampling shifts, and the preservation of original digital recordings facilitate potential external validation. Utilizing a recognized qualitative analysis methodology also improves our interpretive transparency, as does the use of rich quotes.

Addressing transferability, all Swedish healthcare regions were represented, but only 12 individual regions participated in an interview, while two provided written answers, meaning that 7 regions are not represented at all, despite multiple efforts for contact. In fact, it was generally difficult to find professionals willing to be interviewed, probably due to the inherent ambiguities around the state funds – a difficulty that should be considered important data.

## Conclusion

This study uncovered the challenges faced in implementing the Child Health Services Accessibility Agreement due to unclear goals and definitions, funding uncertainties, issues with project prioritization and partner collaboration, and the challenges of staff retention and foundational program stability. Although the agreement’s goals were relevant, these hurdles made them challenging to implement. Given that Swedish governments – irrespective of political colour – have a preference for soft governance through directed state funds, the key takeaways from this study are likely to have continued relevance. These are: the importance of having clearly defined goals and guidelines when implementing funding decisions, consistent funding with a clear timetable, facilitating collaboration across sectors, and selecting areas for extra funding that have a solid foundational support system on the ground.

## Data Availability

The qualitative data supporting the findings of this study are derived from structured interviews. Given the sensitive nature of the interviews and the need to uphold participant confidentiality, the raw data cannot be made publicly accessible. These data management practices are in accordance with the ethical guidelines to ensure the privacy and protection of participant information.Anonymized data excerpts that support the study’s conclusions, along with detailed information on the coding scheme and thematic analysis process, are available upon reasonable request. Access will be granted provided that such inquiries are for legitimate academic purposes and that the requesting party agrees to maintain the confidentiality of the shared data.Interested researchers are encouraged to contact the corresponding author.

## Abbreviations

C-CHCs: Central Child Health Services
SALAR: Swedish Association of Local Authorities and Regions
SES: Socioeconomic Status
CNI: Care Needs Index
OECD: Organization for Economic Co-operation and Development
UNICEF: United Nations International Children’s Emergency Fund
WHO: World Health Organization
EU: European Union
